# International study of 24-h movement behaviors of early years (SUNRISE): a pilot study from Bangladesh

**DOI:** 10.1186/s40814-021-00912-1

**Published:** 2021-09-15

**Authors:** Mohammad Sorowar Hossain, Iztiba M. Deeba, Mahmudul Hasan, Katharina E. Kariippanon, Kar Hau Chong, Penny L. Cross, Shameema Ferdous, Anthony D. Okely

**Affiliations:** 1 Department of Emerging and Neglected Diseases, Biomedical Research Foundation, Dhaka, Bangladesh; 2 Early Start, School of Health and Society, University of Wollongong, Wollongong, NSW Australia

**Keywords:** SUNRISE study, 24-h movement behaviors, COVID-19, Bangladesh

## Abstract

**Background:**

The World Health Organization (WHO) released guidelines for physical activity, sedentary behavior, and sleep for children under 5 years of age in 2019. In response to these guidelines, this pilot study aimed to (i) determine the proportion of preschool children (ages 3-4 years) who met the WHO guidelines; (ii) examine the feasibility of the proposed protocol for the SUNRISE study; and (iii) assess the impact of the COVID-19 pandemic on movement behaviors of preschool children in Bangladesh.

**Methods:**

Time spent in physical activity, sedentary behavior and sleep were objectively measured using two types of accelerometers (ActiGraph wGT3x-BT and ActivPAL4). Screen time and sleep quality were assessed via parent questionnaire. Fine and gross motor skills were measured using the Ages and Stages Questionnaire (3rd edition). Three executive functions were assessed using the Early Years Toolbox. Focus groups were conducted with parents and childcare staff to determine the feasibility of the protocol. Follow-up data during COVID-19 pandemic was collected from parents over phone.

**Results:**

Data from 63 preschool-aged children and their parents was analyzed in this pilot study. Only three children (4.7%) met all components of the WHO guidelines. Separately, children meeting physical activity, sedentary screen time and sleep guidelines were 71.9%, 17.5%, and 59.7% respectively. The proportion of all children who were developmentally on-track for the gross and fine motor skills was 58.7% and 50.8%, respectively. Parents and educators reported that the protocol was feasible except for the activPAL-4 accelerometer. Approximately, 39% of children (14 out of 37) who wore this device developed itchy skin and rashes resulting in the suspension of using this device mid-way through data collection. During COVID-19, there was a significant decrease in children’s total physical activity (− 193 min/day), and time spent outside on weekdays (− 75 min/day) and weekend days (− 131 min/day) and a significant increase in sedentary screen time (+85 min/day).

**Conclusion:**

Only a low proportion of children met the WHO guidelines. Methods and devices (except ActivPAL4) used in this pilot study proved to be feasible and this has paved the way to conduct the main SUNRISE study in Bangladesh. Future measures should be taken to address the issue of movement behaviors of children during the time of pandemics like COVID-19.

**Supplementary Information:**

The online version contains supplementary material available at 10.1186/s40814-021-00912-1.

## Key messages regarding feasibility

1. What uncertainties existed regarding the feasibility?

- The ability to collect data in pre-schools in Bangladesh
- If children would be able to wear the different accelerometers for the required length of time
- If parents were able to validly answer the questions relating to their child’s movement behaviors.

2. What are the key feasibility findings?
  Different methods (such as assessing fine and gross motor skills, three executive functions using Early Year Toolbox) and devices used in this pilot study and the majority of questions in the parent questionnaire were feasible in Bangladesh context. However, the activPAL acclerometer was unsuitable due to the hot and humid climate in Bangladesh.
3. What are the implications of the feasibility findings for the design of the main study?
  We recommend that the waist-worn ActiGraph be used in the SUNRISE main study.

## Introduction

Childhood obesity poses an urgent public health challenge in the twenty first century and is rising at an alarming pace in both high income and low- and middle-income countries (LMICs). In 2019, around 38 million children (age < 5 years) were reported as having overweight or obesity and according to a recent projection, 45% (approximately 4 billion) of the global population will experience obesity by 2035 [1–3].

Not only are immediate health and quality of life affected, but children with obesity are also at greater risk of developing several non-communicable diseases such as diabetes, cardiovascular diseases, hypertension, stroke, and cancer [2, 4]. Obesity at a young age tends to persist into adolescence and adulthood and can give rise to chronic illness and premature death [3].

In Bangladesh, a lower middle-income country, childhood overweight and obesity have increased over the years, from 3.6% in 1998–2003 to 7.9% in 2010–2015 [4]. Currently, the proportion of children with overweight and obesity are overwhelmingly higher in low- and middle-income countries than in high-income countries [5, 6]. In our recent study, we found around 14% children (4–7 years old) were overweight/obese at urban community setting in Bangladesh [7].

Movement behaviors such as low level of physical activity, prolonged sedentary activities and short duration of sleep are well established factors contributing to the development of overweight and obesity in childhood [8]. Though several intervention strategies have been implemented to overcome the obesity epidemic, they typically focused on individual movement behavior while excluding the others [9]. Since these strategies fail to recognize the health implications of the movement behaviors in a holistic way, the importance of developing an integrated approach including guidelines for all the movement behaviors was deemed to be of supreme importance. Thus, in 2019, the World Health Organization (WHO) released global guidelines for physical activity, sedentary behavior, and sleep for children under 5 years of age [10].

The primary goal of The International Study of Movement Behaviors in the Early Years (SUNRISEhttps://sunrise-study.com/) study is to assess the proportion of children adhering to the WHO Global guidelines from countries of different socioeconomic status. In addition to this, it aims to determine how 24-h movement behaviors are linked with overweight and obesity, gross and fine motor skills and executive function in the early years, and the variations among the countries [11]. Prior to conducting the study on a large scale, it was essential to assess the feasibility of the protocol in each participating country. Thus, the SUNRISE pilot study was designed and piloted in Bangladesh, a densely populated LMIC, in March 2019. In this paper, we report the results of the Bangladesh SUNRISE pilot study.

The coronavirus (COVID-19) pandemic created an unprecedented situation impacting on every sphere of personal, social and economic life. To stop the transmission of the SARS-CoV-2, the causative of COVID-19, countries around the world implemented various public health measures (e.g., lockdowns, school closures and movement restrictions). Bangladesh imposed a nationwide lockdown during the initial phase (23 March 2020 to 30 May 2020) of the COVID-19 pandemic which caused widespread panic [12]. The changed environment of the COVID-19 pandemic created a unique opportunity to re-assess how the movement behaviors among children enrolled in the SUNRISE study had changed due to COVID-19.

The objectives of this study were to (i) determine the proportion of preschool children (aged 3 and 4 years) in Bangladesh who met the WHO guidelines for physical activity, sedentary behavior and sleep, (ii) examine the feasibility of the methods to be used in the SUNRISE main study, and (iii) assess the impact of COVID-19 pandemic in the movement behaviors of Bangladeshi preschool children.

## Materials and methods

### Study setting and participants

The SUNRISE pilot study was conducted in three schools in the greater metropolitan area of Dhaka: two schools were in an urban area (Dhaka Premier school and Akij Foundation School) and one (AkijBonoful Foundation School) in a rural-like setting (less-developed part of Dhaka). The principal of each school was contacted, informed, and permission was granted for the study. Parents of eligible children were contacted through school class teachers to participate in the study. Interested parents were gathered in a hall room of the respective school. One team of data collectors interviewed parents while other team was involved with various measurements (height, weight, executive functions, and motor skills) and placing accelerometers on children. The purpose of the study was explained to parents. Once parents provided informed written consent, children aged between 3 and 5 years took part in the study. Each school supported to make an arrangement in case participating child became unsettled during study. The ethical approval (Memo No: BRF/ERB/2019/03) of this study was obtained from the Institutional Review Board of Biomedical Research Foundation, Bangladesh, and the University of Wollongong Human Ethics Research Committee.

The study was conducted between March 2019 and June 2019. All recruited data collectors underwent extensive training before field level data collection. Anthropometric measurements and assessment of fine and gross motor skills, and executive functions of participating children were completed in a single day. An interviewer-administered parent questionnaire (in local language) providing basic demographic information for both parent/caregiver and child and the movement behaviors of their child was completed on the same day. As a follow-up study, parents who had participated in the SUNRISE pilot study were contacted again over the phone between 20 May 2020 and 30 May 2020 during the COVID-19 pandemic to collect data on children’s movement behaviors during the pandemic.

### Measures and procedures

#### Anthropometrics

The height of the children was measured using a portable stadiometer (Leicester 214 Transportable Stadiometer; Seca, Germany) and weight was measured barefoot using a digital scale (SECA 878). All measurements were taken twice and average was used for analysis. Body mass index (BMI) and associated *z*-scores for BMI-for age (BAZ), height-for-age (HAZ), and weight-for-age (WAZ) were computed using the WHO AnthroPlus software (version 1.0.4; WHO, Geneva, Switzerland). Children were classified according to the reference standards of the WHO [13, 14].

#### Accelerometry

To assess the physical activity, sedentary time, and sleep patterns of children, two types of accelerometers were used: the ActiGraph wGT3x-BT was worn over the right hip and the activPAL 4 was attached on the right thigh before taking other measurements. The children were asked to wear the accelerometers for 4 days. They could remove the ActiGraph only while bathing or swimming thus preserving the monitor from water.

The ActiLife software (version 6.1.2.1, ActiGraph Corporation) was used to initialize, download, and process the ActiGraph data. Accelerometers were programmed to capture data at a sampling rate of 30 Hz, which was integrated into 15-s epochs for the analysis using the low-frequency filter. To be included in the analyses, a child needed to have at least 24 h of valid data, with a minimum of 6 h of valid wear time during waking hours. The identification of valid 24hr day(s) was done through visual inspection of the ActiGraph data to confirm if there were movement peaks throughout the entire day (defined as midnight to midnight). If there were little to no peaks throughout the sleeping period, it was assumed that the child took off the monitor during the particular day(s) and was not included in the subsequent analyses. A pre-determined time filter (i.e., 7:20 AM to 21:10 PM) (based on the average parent-reported wake-up and bedtime of the study population) was applied to all valid 24 h day(s) to exclude sleeping period from the analyses of physical activity (PA) and sedentary behavior (SB). Further, any waking periods with => 20 consecutive minutes of zero counts were defined as non-wear and were excluded from the analysis [15]. The cut-points were used to classify valid waking wear time as SB (< 800 counts per minute [CPM]), light-intensity PA (LPA) (800–1679 CPM), moderate-intensity PA (MPA) (1680–3367 CPM) or vigorous-intensity PA (VPA) (=> 3368 CPM) [16, 17]. Due to skin issues with the activPAL, only the feasibility of the activPAL was assessed and reported in this study.

#### Sleep, screen-time, and restrained sitting

Caregivers were asked to report their child’s nighttime sleep and nap schedules. Sleep duration was calculated as the sum of nighttime sleep (difference between bedtime and wake-up time) and nap duration. Caregivers also reported time spent outside, screen time and use of screens before bedtime; time spent restrained (strapped in and unable to move), and time spent sitting.

#### Executive functions

Executive function tests were performed to assess the cognitive flexibility (shifting), control of behavioral urges and impulses (inhibition), and visual-spatial working memory of children [18]. Three personalized iPad games (www.eytoolbox.com.au) including “Card Sort”, the “Go/No-Go”, and the “Mr. Ant” [18]. It took approximately 5 min to complete the “Card Sort” game and the total scores ranged between 0 and 12 points [18]. This game measured the ability to flexibly shift attention. “Go/No-Go” was designed to assess inhibitory control. On the iPad screen, a fish or a shark appeared randomly, and the children needed to tap the fish but not the shark before it quickly passed. The scores for this game ranged between 0 and 1, with 1 representing the highest score [18]. The score was produced by calculating the proportion of fishes caught multiplied by the proportion of sharks spared by the children. “Mr. Ant” assessed the child’s working memory. In this game an ant appears on screen with a number of colored dots placed in different spatial locations on its body. The dots vanish after some time, and the child needs to recall and tap the locations of the dots. The score was provided after the completion of each level and ranged between 0 and 8 points [18].

#### Gross and fine motor skills

The Ages and Stages Questionnaire-3 (48 months, ASQ3) was used to assess gross and fine motor skills [19]. Participating children got the opportunity to attempt tasks twice and their performance was categorized as “yes”, “sometimes”, or “not yet”. If children were able to perform the task on a single attempt, they received ‘yes’. If they initially failed, they got a second attempt and received a “sometimes” if successful or “not yet” if they failed both attempts. For fine motor skills assessment, children had one chance to complete the tasks and were classified as either “yes” or “not yet” and scored according to the ASQ-3 tool. For each ASQ task the child was classified as (i) child requires follow-up and further assessment/action; (ii) child is developing on schedule but may benefit from extra practice in some of the skills; (iii) child is developing on schedule [19].

### COVID-19 questionnaire

The COVID-19 questionnaire consisted of 25 questions relating to the children’s physical activity, sedentary behavior, sedentary screen time, and sleep at the height of COVID-19 restrictions in Bangladesh. The questions regarding time spent in various movement behaviors and time spent outdoors were identical to the previous questionnaire.

#### Sample size

According to the SUNRISE study protocol, the primary aim of the pilot study was to determine the feasibility of recruiting children from urban and rural settings, and to test the feasibility and acceptability of the data collection measures proposed for the main study. It was determined that a sample of 100 children, 50 each from a rural and urban location, would be sufficient [11]. .

### Data analysis

All statistical analyses were performed using SPSS Statistics for Window version 26.0 (IBM Corp, Armonk, NY). Descriptive statistics (mean and 95% confidence interval (CI) or frequency and percentage) were computed for all study variables. Mann-Whitney *U* tests were conducted to examine differences in anthropometric indicators, movement behaviors, motor skills and executive function between the sexes and residential settings. Chi-square tests or Fisher exact tests were used to examine differences in the proportion of children meeting the 24-h movement guidelines between the sexes. Wilcoxon-signed rank tests were used to examine changes in movement behaviors before and during COVID-19 period. Statistical significance was set at *p* < 0.05. All hypothesis testing should be treated as preliminary and treated with caution due to the study being underpowered.

## Results

A total of 65 preschoolers participated with 48 from urban and 19 from less-developed areas. The mean age of 4.5 years and 55.4% of children were male.

Table 1 shows anthropometric outcomes and the amount of time spent in the different movement behaviors, stratified by sex and urban/less-developed setting. No significant differences were found between girls and boys for any of the anthropometric indicators. However, children from less-developed setting had significantly lower BMI (14.3 [95%CI 13.7, 14.8] vs. 15.8 [95%CI 15.3, 16.3]) and BAZ (− 0.85 [95%CI − 1.31, − 0.39] vs. 0.30 [95%CI − 0.02, 0.62]) scores than their urban counterparts. No significant differences between sexes were found for the amount of time spent in any of the movement behaviors.

**Table 1:** Descriptive characteristics and time spent in movement behaviors of participating children, stratified by sex and residential settings

Variables	All (***n*** = 64)	Boys (***n*** = 36)	Girls (***n*** = 28)	***p*** value^**d**^	Urban (***n*** = 49)	Less developed (***n*** = 15)	***p*** value^**e**^
Age	4.5 (4.4, 4.7)	4.6 (4.4, 4.7)	4.5 (4.3, 4.7)	0.72	4.5 (4.4, 4.6)	4.7 (4.3, 5.1)	0.60
Weight (kg)	17.3 (16.5, 18.0)	17.3 (16.2, 18.3)	17.3 (16.2, 18.4)	0.96	17.6 (16.7, 18.4)	16.3 (14.9, 17.7)	0.27
Height (cm)	105.5 (104.0, 107.1)	105.6 (103.2, 107.9)	105.5 (103.5, 107.6)	0.90	105.2 (103.5, 106.9)	106.6 (102.4, 110.7)	0.16
BMI	15.4 (15.0, 15.8)	15.4 (14.9, 15.9)	15.5 (14.8, 16.2)	0.97	15.8 (15.3, 16.3)	14.3 (13.7, 14.8)	**0.001**
HAZ	− 0.24 (− 0.57,0.09)	− 0.32 (− 0.82, 0.17)	− 0.13 (− 0.56, 0.29)	0.69	− 0.26 (− 0.60, 0.07)	− 0.16 (− 1.12, 0.79)	0.54
WAZ	− 0.14 (− 0.45,0.17)	− 0.21 (− 0.66, 0.24)	− 0.05 (− 0.48, 0.39)	0.58	0.03 (− 0.31, 0.36)	− 0.69 (− 1.42, 0.05)	0.12
BAZ	0.03 ± (− 0.26, 0.32)	0.02 (− 0.36, 0.39)	0.05 (− 0.42, 0.52)	0.87	0.30 (− 0.02, 0.62)	− 0.85 (− 1.31, − 0.39)	**0.001**
LPA (min/day)^a^	93.6 (86.3, 100.9)	92.9 (84.0, 101.7)	94.5 (81.4, 107.5)	0.79	87.0 (79.5, 94.6)	111.8 (95.9, 127.8)	**0.006**
MPA (min/day)^a^	64.0 (56.9, 71.2)	64.2 (55.1, 73.3)	63.9 (51.8, 75.9)	0.96	57.7 (50.5, 65.0)	81.7 (65.5, 97.9)	**0.024**
VPA (min/day)^a^	19.6 (16.6, 22.7)	20.6 (16.3, 24.9)	18.5 (13.8, 23.2)	0.54	18.2 (14.9, 21.5)	23.8 (16.3, 31.2)	0.19
MVPA (min/day)^a^	83.7 (73.8, 93.6)	84.8 (71.8, 97.7)	82.3 (66.0, 98.7)	0.89	75.9 (65.8, 86.1)	105.4 (82.3, 128.6)	**0.040**
TPA (min/day)^a^	177.3 (160.8, 193.8)	177.6 (157.1, 198.2)	176.8 (148.1, 205.5)	0.82	163.0 (146.0, 179.9)	217.3 (180.2, 254.4)	**0.011**
SB (min/day)^a^	572.3 (553.9, 590.7)	567.5 (541.9, 593.0)	578.5 (550.3, 606.6)	0.54	587.3 (569.4, 605.2)	530.4 (484.0, 576.7)	**0.020**
SST (min/day)^b^	160.9 (138.4, 183.5)	163.3 (134.2, 192.5)	157.8 (120.1, 195.4)	0.61	175.5 (149.0, 202.1)	114.3 (77.9, 150.7)	**0.029**
Sleep (min/day)^c^	607.8 (588.2, 627.5)	610.6 ± (585.4, 635.7)	604.0 (570.7, 637.4)	0.81	611.4 (589.8, 633.0)	595.7 (544.5, 646.9)	0.36

*BMI* body mass index, *LPA* light-intensity physical activity, *MVPA* moderate-vigorous intensity physical activity, *TPA* total physical activity, *SB* sedentary behavior, *SST* sedentary screen time

Data presented as mean (95% confidence interval)..

Analytical sample: ^a^*n* = 57 (32 boys, 25 girls; 42 urban, 15 less developed)

^b^*n* = 63 (36 boys, 27 girls; 48 urban, 15 less developed)

^c^*n* = 62 (36 boys, 26 girls; 48 urban, 14 less developed)

Differences were tested using Mann-Whitney *U* tests by ^a^sex and ^b^residential settings; bold values indicate statistically significant at *p* < 0.05

There were differences in time spent in movement behaviors between the children from urban and less-developed setting. Children from the less-developed setting spent a significantly higher amount of time in LPA (111.8 [95%CI 95.9, 127.8] vs. 87.0 [95%CI 79.5, 94.6]), MVPA (105.4 [95%CI 82.3, 128.6] vs. 75.9 [95%CI 65.8, 86.1]), and total PA (i.e., sum of LPA and MVPA) (217.3 [95%CI 180.2, 254.4] vs. 163.0 [95%CI 146.0, 179.9]) compared to urban children. Conversely, urban children spent significantly greater amount of time in SB (587.3 [95% CI 569.4, 605.2] vs. 530.4 [95% CI 484.0, 576.7]) and sedentary screen time (175.5 [95%CI 149.0, 202.1] vs. 114.3 [77.9, 150.7]) than children from less-developed setting. Most children performed screen-based activities just before sleep (95.2%, *n* = 60) and had screen device in bedroom (74.2%; *n* = 46).

The proportions of children meeting the WHO guidelines are shown in Table 2. Only three children (4.7%) met the all components of the guidelines. The proportion of children who met the physical activity, sedentary screen time, and sleep guidelines were 71.9% (*n* = 41), 17.5% (*n* = 11), and 59.7% (*n* = 37), respectively. There were no statistical differences between boys and girls in terms of meeting and not meeting guidelines individually or combined.

**Table 2:** Number and proportion of children meeting each of the 24-h movement guidelines, stratified by sex

Variables	Total ***n*** (%)	Boys ***n*** (%)	Girls ***n*** (%)	***p*** value^**e**^
≥ 60 min/day of MVPA per day^a^	41 (71.9)	23 (71.9)	18 (72.0)	0.99
≥ 180 min/day of TPA^a^	24 (42.1)	14 (43.8)	10 (4.0)	0.78
≥ 60 min/day of MVPA and ≥ 180 min/day of TPA^a^	24 (42.1)	14 (43.8)	10 (4.0)	0.78
≤ 60 min/day of SST^b^	11 (17.5)	7 (19.4)	4 (14.8)	0.75
10–13 h/day of sleep^c^	37 (59.7)	22 (61.1)	15 (57.7)	0.79
Meeting all five recommendations	3 (4.7)	3 (9.4)	0	*N/A*

*MVPA* moderate-vigorous intensity physical activity, *TPA* total physical activity, *SST* sedentary screen time

Analytical sample: ^a^*n* = 57 (32 boys, 25 girls); ^b^*n* = 63 (36 boys, 27 girls); ^c^*n* = 62 (36 boys, 26 girls); ^d^*n* = 55 (32 boys, 23 girls)

^e^Differences between sexes were tested using Pearson chi-square tests or Fisher exact tests; *N/A* no statistical test was conducted due to the zero proportion identified for girls

The results of the gross and fine motor skills tasks and executive functions were presented in Table 3. Overall, no difference between boys and girls or between children of urban and less-developed areas was observed for any motor skill tasks. The proportion of all children who were developmentally on-track for the gross and fine motor skills were 58.7% (*n* = 37) and 50.8% (*n* = 32), respectively. No significant differences were observed between boys and girls and for any of the executive function tasks; however, children from less-developed setting had significantly higher working memory scores than their urban counterparts (2.26 [95%CI 1.77, 2.76] vs. 1.58 [95%CI 1.31, 1.85]).

**Table 3:** Motor skills and executive functions of participating children, stratified by sex and residential settings

Variables	All (***n*** = 63)	Boys (***n*** = 35)	Girls (***n*** = 28)	***p*** value^**d**^	Urban (***n*** = 48)	Less developed (***n*** = 15)	***p*** value^**e**^
Gross motor skills	46.7 (43.4,50.0)	46.1 (41.4,50.9)	47.5 (42.6, 52.3)	0.78	45.7 (41.6, 49.8)	50.0 (44.8, 55.2)	0.42
Development status, n (%)							
Below cutoff Close to cutoff Above cutoff	17 (27.0) 9 (14.3) 37 (58.7)	10 (28.6) 6 (17.1) 19 (54.3)	7 (25.0) 3 (10.7) 18 (64.3)		15 (31.3) 5 (10.4) 28 (58.3)	2 (13.3) 4 (26.7) 9 (60.0)	
Fine motor skills	40.0 (35.7, 44.3)	36.3 (29.9, 42.6)	44.6 (39.0, 50.3)	0.07	39.4 (34.3, 44.4)	42.0 (32.7, 51.3)	0.58
Development status, n (%)							
Below cutoff	14 (22.2)	11 (31.4)	3 (10.7)		11 (22.9)	3 (20.0)	
Close to cutoff	17 (27.0)	9 (25.7)	8 (28.6)		14 (29.2)	3 (20.0)	
Above cutoff	32 (50.8)	15 (42.9)	17 (60.7)		23 (47.9)	9 (60.0)	
Executive functions							
Working memory^a^	1.74 (1.50, 1.98)	1.68 (1.34, 2.03)	1.81 (1.47, 2.16)	0.61	1.58 (1.31, 1.85)	2.26 ± (1.77, 2.76)	**0.024**
Inhibition^b^	0.48 (0.42, 0.54)	0.49 (0.40, 0.59)	0.46 (0.38, 0.54)	0.69	0.46 (0.39, 0.53)	0.53 (0.39, 0.67)	0.24
Shifting^c^	7.15 (6.55, 7.75)	7.00 (6.16, 7.84)	7.33 (6.44, 8.23)	0.45	7.30 (6.57, 8.02)	6.64 (5.59, 7.70)	0.34

Data presented as mean(95% confidence interval), unless otherwise indicated.

Analytical sample: ^a^*n* = 60 (35 boys, 25 girls; 46 urban, 14 less developed); ^b^*n* = 51 (27 boys, 24 girls; 37 urban, 14 less developed); ^c^*n* = 61 (34 boys, 27 girls; 47 urban, 14 less developed )

Differences were tested using Mann-Whitney *U* tests by ^d^sex and ^e^residential settings; bold values indicate statistically significant at *p* < 0.05

Most caregivers who consented to participate in the follow-up study during the COVID-19 pandemic were concerned about their child’s physical activity levels (66.7%; *n* = 26), sedentary screen time (74.4%; *n* = 29), and sleep (51.3%; *n* = 20), and nearly half felt they could not support their child to accrue a healthy balance of movement behaviors (46.2%; *n* = 18). Table 4 presents parent-reported physical activity, time spent outside, and screen time pre-COVID-19 and during the pandemic. Most importantly, during the COVID-19 pandemic there was a significant decrease in Bangladeshi children’s total physical activity (− 193 min/day), and time spent outside on weekdays (− 75 min/day) and weekend days (− 131 min/day). On the other hand, sedentary screen time of children increased significantly (+85 min/day) during the COVID-19 pandemic.

**Table 4:** Comparison of parent-reported physical activity, sedentary screen time, sleep duration, and time spent outside between pre- and during COVID-19

Variables	Pre-COVID-19	During COVID-19	***p*** value^**a**^
TPA (min/day) (*n* = 33)	270.3 (219.5, 321.1)	77.3 (54.1, 100.5)	**< 0.0001**
MVPA (*n* = 35)	83.7 (54.5, 112.9)	25.4 (16.7, 34.1)	**< 0.0001**
Sleep (min/day) (*n* = 30)	605.5 (576.7, 634.4)	593.0 (577.8, 608.2)	0.33
SST (min/day) (*n* = 34)	192.2 (160.9, 223.5)	277.0 (227.3, 326.8)	**0.005**
Time spent outside weekdays (min/day) (*n* = 33)	95.3 (61.4, 129.2)	20.9 (3.8, 38.0)	**0.0002**
Time spent outside weekend days (min/day) (*n* = 33)	154.2 (108.6, 199.9)	23.6 (6.5, 40.8)	**< 0.0001**

*MVPA* moderate-vigorous intensity physical activity, *TPA* total physical activity, *SST* sedentary screen time

Data presented as mean (95% confidence interval)

^a^Differences were tested using Wilcoxon signed rank tests; bold values indicate statistically significant at *p* < 0.05

Analysis of the protocol feasibility revealed the following regarding compliance with the 24 h accelerometer protocol: 89% (*n* = 57) of children wore the monitors for a minimum of 1 day (i.e., provided at least 24-h data; 47 [82.5%] provided 3 days of 24-h data); others (11%; *n* = 7) removed the monitors during the night sleep time and/or did not wear the monitor for a significant portion of waking time. On the other hand, more than half of these children (57.8%; *n* = 37) also wore the activPAL devices simultaneously. However, 37.8% of them (*n =* 14) developed itchy skin/rashes after wearing the devices. As a result, it was determined to suspend the collection of movement behavior data using the activPAL monitor mid-way through the data collection period. Participation in the executive function tasks: 79.7% (*n* = 51) completed the inhibition task, 93.7% (*n* = 60) completed the working memory task, and 95.3% (*n* = 61) completed the shifting task.

## Discussion

To the best of our knowledge, this was the first study of its kind to investigate the 24-h movement behaviors quantitatively irrespective of age groups of children in Bangladesh. Overall, a low proportion of preschool children met the WHO Global guidelines. We found significant differences in movement behaviors and working memory between urban and children from less-developed setting. During the nationwide lockdown due to the COVID-19 pandemic, physical activity was severely reduced, while sedentary screen time among Bangladeshi preschoolers. Implementation of the SUNRISE protocol was found to be feasible in Bangladesh with the exception of the activPAL.

### Proportion of preschool children meeting WHO global guidelines in Bangladesh

In this SUNRISE pilot study in Bangladesh, the proportion (4.7%) of children meeting all components of WHO Global guidelines was lower as compared to studies conducted in Sweden (19.4%, obtained from SUNRISE pilot data), Canada (12.7%), Australia (14.9%), China (15%), and Belgium (5.6%) [5, 20–22]). The proportion of Bangladeshi children meeting the total physical activity recommendation was ~ 41% while a high proportion of Swedish (~ 90%) South African (84%, obtained from SUNRISE pilot study), Australian (~ 90%), and Chinese (65.4%) children met the physical activity guidelines [5, 21–23]. Variations in time spent on physical activity guideline could be associated with methodical differences in the studies. For Bangladeshi children, both ActiGraph and ActivPAL acceleromoters were used while other studies used ActiGraph.

In Bangladesh, the proportion of children meeting the sedentary screen time recommendation was found to be 17.5%. Our result is similar to Australia but lower than Sweden (37.8%) and South Africa (48%). A lower proportion (59.7%) of Bangladeshi children met sleep recommendation as compared to children in Sweden (62.5%), Canada (83.9%), Australia (88.7%), and South Africa (66%), while only 29.5% of children met the sleep recommendation [15, 20, 21, 23].

The lower physical activity and higher screen time in Bangladesh could be attributed to lack of adequate playgrounds in schools and neighborhoods in Dhaka, which is a highly crowded (> 47,000/km^2^) megacity, with a population in excess of 20 million [24]. Bangladesh is undergoing rapid and unplanned urbanization, with playgrounds for children and green spaces for outdoor play largely ignored in urban planning. Unlike in many HICs, most families in Dhaka City live in small apartments where children share a bed with their parents or siblings. As expected, screen devices were present in majority of bedrooms of the children and most of them used screens just before sleep.

### Feasibility of SUNRISE protocol in Bangladesh context

Considering the feasibility and willingness of children to wear the devices, the ActiGraph yielded better results compared to the activPAL. A high compliance for the ActiGraph (only 7 children refusing to wear the monitor during the sleep) and a low compliance for activPAL was observed (27 children refused to wear altogether). Further, since 37.8% (14) children developed itchy skin and rashes after wearing the activPAL, this device seems unsuitable for use in countries with hot and humid climates, such as Bangladesh. Thus, we recommend that the ActiGraph is used in the SUNRISE main study to increase compliance in hot and humid countries.

We found that the gross and fine motor skill assessments were feasible and acceptable in the Bangladesh context. Bangladeshi children performed poorly in gross and fine motor skills tasks (46.7 ± 13.1 and 40.0 ± 17.2 points) compared to the children from developed countries like Norway (54 ± 9 and 50 ± 13 points) and America (52 ± 10 and 44 ± 14 points) [25]. Here, the gap between the scores might be due to the different methodology used in those studies. In the Norwegian and American studies, the gross and fine motor skills were reported by parents unlike the SUNRISE study where trained research personnel assessed the tasks. On the other hand, Bangladeshi children were quite ahead in points from the Swedish children (41.7 ± 15.6 and 35.5 ± 17.9 points) in terms of both gross and fine motor skills [21]. In Bangladesh, children from the less-developed area demonstrated greater gross and fine motor skills which were significantly higher than the urban children and happened to engage more in physical activity in open spaces available there. It is well established that children’s cognitive development is associated with fulfilling the minimum requirement of physical activity and findings from Bangladesh SUNRISE pilot study supported this theory [26, 27].

The executive function protocol was feasible among the children. While assessing the executive functions, children started with “Mr. Ant” and then completed the “Go/No-Go”. The “Card Sort” task was the last to be tested with a sufficient break as most children could not hold the concentration for long. Children from less-developed area played the “Card Sort” game in a separate day with a fresh start. Children irrespective of area they were from performed the best in the Mr. Ant task and the least well in the “Card Sort” task.

### Impact of COVID-19 pandemic on 24-h movement activities in Bangladesh

Pandemics such as COVID-19 can influence the movement behaviors significantly, due to the restrictions placed on movement and the closure of preschools. In our study, children’s physical activity was reduced more than threefold, while screen time increased significantly. The opposite results for physical activity were observed in Swedish children during COVID-19 pandemic (ref). This was because of differences in public health policies set by the two countries. In Bangladesh, lockdown was strictly maintained during the early phase of the pandemic and people were so panicked that most people in Dhaka City were stayed home. Unlike the situation in many high-income countries, there are no backyards or parks in Dhaka City and therefore there was limited scope for physical activity for children in Bangladesh. In future potential infectious disease outbreaks, the issue of physical activity needs to be addressed by the policymakers of the country.

## Strengths and limitations

The device-based measurement of 24-h movement behaviors (physical activity, sleep), gross and fine motor skills and executive functions of individual preschool-aged children in LMIC setting like Bangladesh is the major strength of this SUNRISE pilot study. On the other hand, convenient sampling and the small sample size are the major limitations of this study and therefore results from this study would not be generalizable to Bangladesh [28].

## Conclusion

This SUNRISE Bangladesh pilot study provided initial findings of 24-h movement behaviors of preschool-aged children in Bangladesh. Only 4.7% of Bangladeshi preschool-aged children met the WHO Global guidelines. A low proportion of children met physical activity and screen time guidelines. During COVID-19 pandemic, the children’s physical activity and time spent outside decreased but screen time was increased significantly in Bangladesh. Future studies with a representative sample size are needed to confirm these results and investigate associated factors. As the methods and devices used in this pilot study were proved to be feasible in Bangladesh setting, it has paved the way to conduct the main SUNRISE study in Bangladesh to depict the real scenario of children meeting WHO Global guidelines.

## Supplementary Information

**Additional file 1.**

## Data Availability

Available upon request to the SUNRISE team.
