# Supplementary material for: International study of 24-h movement behaviors of early years (SUNRISE): a pilot study from Bangladesh
Source: Pilot Feasibility Stud. 2021 Sep 15;7:176. doi: 10.1186/s40814-021-00912-1 (PMC8440144; doi:10.1186/s40814-021-00912-1)
Supplement: Supplementary file 1 — Additional file 1. [file 40814_2021_912_MOESM1_ESM.docx]

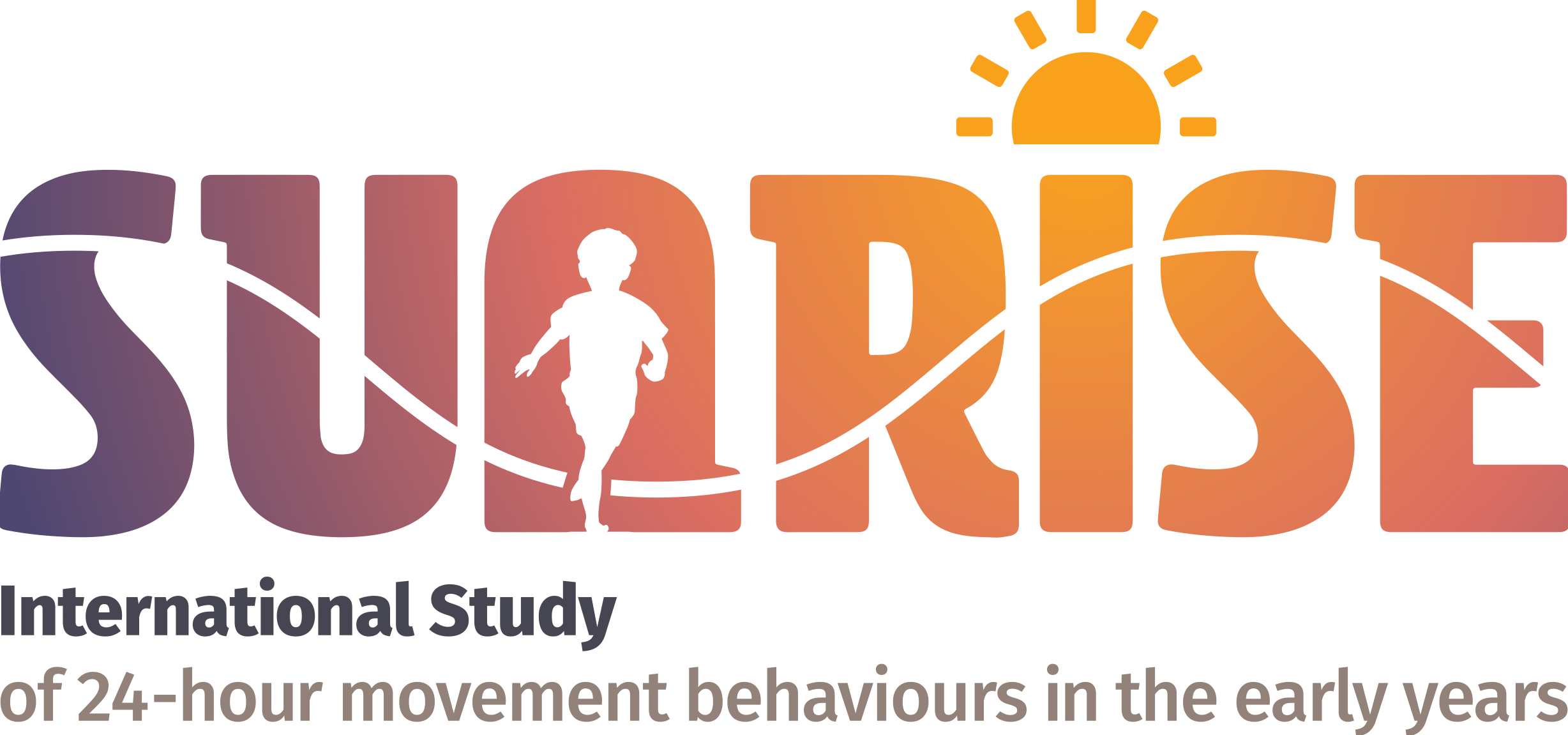


Office use only

Child ID: __ __ __ __ __ __ __

**THE SUNRISE STUDY**

**INTERNATIONAL STUDY OF 24-HOUR MOVEMENT BEHAVIOURS IN THE EARLY YEARS**

**PARENT/CAREGIVER QUESTIONNAIRE**

*This questionnaire is to be administered/completed by the MAIN caregiver of the child who lives with them and has previously completed this questionnaire. A separate questionnaire should be used for each eligible child who participated.*

This survey asks you about your child’s movement behaviours (physical activity, sedentary behaviour, screen time and sleep) **during COVID-19 related restrictions in your country.** If restrictions have been lifted or minimized, we ask you to consider your child’s movement behaviours for a typical week when **all** restrictions were in place.

| **CHILD AND CAREGIVER BACKGROUND** | |
| --- | --- |
| **1.** | Parent/caregiver's relationship to the child participating in the study? |
|  | Mother  Father  Grandmother  Grandfather  Legal Guardian  Other (please specify): ……………………………………………….. |
| **2.** | Was your child sick in the last week, or did anything prevent [him / her] from being active at home in the last week?  🞏 Yes 🞏 No  **If yes, go to 2a** |
| **2a.** | **If Yes,** what prevented your child from being active?  ___________________________________________ |

| **PHYSICAL ACTIVITY, SEDENTARY BEHAVIOUR, SCREEN TIME AND SLEEP** | | |
| --- | --- | --- |
| The next questions ask about your child's movement behaviours during COVID-19 related restrictions. Please report the **number of hours and minutes per day** (for all the questions). E.g. 1 hour and 30 mins | | |
| **3.** | In a typical 24 hour period during COVID-19 restrictions, how much time did the child who is participating in this study spend: (Please record these as accurately as you can to the nearest minute) | |
| **3a.** | In a variety of physical activities, spread throughout the day?  For example: active play, running, playing with balls, moving to music/dancing, swimming, riding a scooter/tricycle/bike. | …..…..hrs ……....mins |
| **3b.** | In energetic play that causes him/her to 'huff and puff' and increases his/her heart rate (this would be part of the total time spent in a variety of activities just mentioned). | …..…..hrs ……....mins |
| **3c.** | Using any electronic screen device such as a smart phone, tablet, video game, or watching television or movies, videos on the internet while they were sitting or lying down? | …..…..hrs ……....mins |
| **4.** | On a typical **weekday** during COVID-19 restrictions, how much time did this child spend outside? | …..…..hrs ……....mins |
| **5.** | On a typical **weekend day** during COVID-19 restrictions, how much time did this child spend outside? | …..…..hrs ……....mins |
| **6.** | In a typical week during COVID-19 restrictions, did your child use electronic screen devices (e.g. TV, video game, computer, tablet or smartphone) in the 2 hours before bedtime on a daily basis?  **If no, go to question 7** | |
|  | Yes  No  Don’t know | |
| **6a.** | **If Yes**, how close to bedtime did your child usually use these devices? | |
|  | Closer than 30 minutes before bedtime  30 mins to less than 1 hour before bedtime  Between 1 and 2 hours before bedtime | |
| **7.** | In a typical week during COVID-19 restrictions, did your child have electronic screen devices in the room where he/she sleeps (e.g. TV, video game, computer, tablet or smartphone)? | |
|  | Yes  No | |
| **8.** | How many hours of sleep did your child get in a typical 24-hours day (**including naps**) during COVID-19 restrictions? | …..…..hrs ……....mins |
| **9.** | In a typical week during COVID-19 restrictions, did your child nap? | |
|  | Yes  No  **If no, go to 10** | |
| **9a.** | What time did your child nap? | |
|  | Begin time: ……………………. End time: ……………………… | |
| **10.** | In a typical week during COVID-19 restrictions, did the child have a consistent bedtime? | |
|  | Yes, bedtime did not vary by more than 30 minutes each day  No, bedtime varied more than 30 minutes each day | |
| **11.** | In a typical week during COVID-19 restrictions, did your child have a consistent wake-up time? | |
|  | Yes, wake-up time did not vary by more than 30 minutes each day  No, wake-up time varied more than 30 minutes each day | |
| **12a.** | In a typical week during COVID-19 restrictions, what time did your child go to bed at night? | |
|  | Bed time: ……………………. PM | |
| **12b.** | In a typical week during COVID-19 restrictions, what time did your child get up in the morning? | |
|  | Wake-up time: ……………………… AM | |
| **13.** | On a scale of 1 to 7, with the higher number indicating higher quality, how would you rate the quality of this child's sleep **in a typical week during COVID-19 restrictions**?  **1 would indicate** very difficult to settle, wakes many times during the night for prolonged periods and is very restless (tosses and turns, throw off bedclothes) **while 7 would indicate** settles and drifts off to sleep within a few minutes, sleeps right through the night, and has a very sound, deep sleep) | |
|  | 1  2  3  4  5  6  7  Don’t know | |

| **COVID-19:** | | | |  |
| --- | --- | --- | --- | --- |
| **14.** | **Due to the impact of COVID-19**, how long has your child stayed at home without going to their childcare centre/pre-school/kindergarten? | | | |
|  | Less than one week  1 to 4 weeks  5 to 8 weeks  More than 8 weeks | | | |
| **15.** | | **If you are/were allowed to go outside during COVID-19 restrictions**, did your child play/go: **(tick as many as appropriate)**  On the property (ie. Garden/yard)  To a friend/relative’s home  To a park/square or playground  To a swimming pool/creek/river/dam/waterhole  To the street  To walk a pet  Any other not mentioned? (please specify)  …………………………………………………………………………………………………….…………………………………………………………  None | |  |
| **16.** | | **During COVID-19 restrictions**, have you been concerned about the level of physical activity, sitting (including screen time) or sleep your child participates in? | |  |
|  | | Physical activity 🞏 Yes 🞏 No  Sitting (including screen time) 🞏 Yes 🞏 No  Sleep 🞏 Yes 🞏 No | |  |
| **17.** | | Do you feel able to support your child to have healthy movement behaviours? | |  |
|  | | 🞏 Yes 🞏 No  **If yes, go to 17a. If no, go to 17b.** | |  |
| **17a.** | | **If yes,** how ?_______________________ | |  |
| **17b.** | | **If no,** why­­­­­­­­­­­­­­­­­­? ______________________ | |  |
| **18.** | | In what type of housing do you and your child live? | |  |
|  | | Low rise apartment/condominium  High rise apartment/condominium  Townhouse  Semi-detached house  Detached house  Attached tube house with/without yards  Other (please specify): ……………………………………………….. | |  |
| **19.** | | How many children (under 18 years old), do you have living in this household **during COVID-19 restrictions**? | |  |
|  | | 1  2  3  4  5  6  7  8  9  10  More than 10 | |  |
| **20.** | | How many adults (18 years old or older, including yourself), do you have living in this household **during COVID-19 restrictions**? | |  |
|  | | 1  2  3  4  5  6  7  8  9  10  More than 10 | |  |
| **21.** | | What are the current working arrangements for your family? (tick all that apply) | |  |
|  | | Mother working at home  Father working at home  Mother going to work as normal  Father going to work as normal  Neither mother or father working  Other (please specify): …………………….. | |  |
| **22.** | | During a typical week under COVID-19 restrictions, how **stressed** did you feel compared to before the restrictions? | |  |
|  | | Less stressed 🞏 About the same 🞏 More stressed | |  |
| **23.** | | During a typical week under COVID-19 restrictions, how **exhausted** did you feel compared to before the restrictions? | |  |
|  | | Less exhausted 🞏 About the same 🞏 More exhausted | |  |
| **24.** | | What kind of support have you received from the childcare centre **during COVID-19 restrictions**?? (tick all that apply) | |  |
|  | | 🞏 A message on prevention of COVID-19 🞏 Story time via audio or video 🞏 Games, for example, structured physical activity games 🞏 Other (please specify) ……………………… 🞏 None | |  |
| **25.** | | What existing resources have you used to support/facilitate/monitor your child’s physical activity and/or screen time at home **during COVID-19 restrictions**?? |  |  |
|  | | 🞏 Smart phone/iPad/similar device apps   - list all the apps that you have used: __________________________________________________________________________________________________________________________________________________________   🞏 Television 🞏 Internet 🞏 Radio 🞏 Other ……………………… 🞏 None |  |  |
| **26.** | | Other comments:  ____________________________________________________________________________________________________________________________________________________________________________________________________________________________________________________________  ____________________________________________________________________________________ |  |  |
| **27.** | | Date survey was completed:  DD/MM/YYYY ______/________/____________ |  |  |
